# A Staff-Directed Electronic Medical Record Alert to Increase Chlamydia Screening: A Randomized Clinical Trial

**DOI:** 10.1001/jamanetworkopen.2026.15360

**Published:** 2026-05-29

**Authors:** Harold C. Wiesenfeld, Jaeyoung Hong, Tong Xu, Kendra M. Cuffe, Kyle T. Bernstein, Thomas L. Gift, Gary S. Fischer

**Affiliations:** 1 Department of Obstetrics, Gynecology, and Reproductive Sciences, University of Pittsburgh School of Medicine, Pittsburgh, Pennsylvania; 2 Magee-Womens Research Institute, Pittsburgh, Pennsylvania; 3 Division of STD Prevention, National Center for HIV, Viral Hepatitis, STD, and Tuberculosis Prevention, Centers for Disease Control and Prevention, Atlanta, Georgia; 4 Division of General Internal Medicine, Department of Medicine, University of Pittsburgh School of Medicine, Pittsburgh, Pennsylvania

## Abstract

**Importance:**

Chlamydia is the most commonly reported sexually transmitted disease (STD) in the US. Screening sexually active females 24 years or younger is recommended to prevent pelvic inflammatory disease and its sequelae; however, screening remains suboptimal.

**Objective:**

To assess whether an automated electronic medical record alert directed at medical assistants who are preparing patients for office visits increases chlamydia screening.

**Design, Setting, and Participants:**

The STD Testing in Outpatient Practices (STOP STDs) Study was a cluster randomized clinical trial conducted from August 2017 to November 2019 (data analyzed from August 2022 to July 2023). The study included medical staff in primary care and obstetrics-gynecology offices across western Pennsylvania.

**Interventions:**

Offices were randomized in a 1:1 ratio to receive a real-time electronic alert or usual care. The alert activated for female patients without chlamydia screening within the past year. Medical assistants received the alert while preparing patients for their visit in an examination room.

**Main Outcomes and Measures:**

The main outcome was the number of chlamydia tests ordered analyzed using constrained difference-in-differences mixed-effects logistic regression with random intercepts for practice and patient; no additional covariates were included.

**Results:**

There were 7356 encounters in primary care and 10 672 in obstetrics-gynecology (women aged 18-24 years). Chlamydia tests were more frequently ordered among patients in primary care practices randomized to the alert (497 of 3770 [13.2%]) than in control practices (135 of 3586 [3.8%]). After adjusting for baseline differences in chlamydia screening in practices, the alert was associated with an increase in test orders compared with the control group (adjusted odds ratio [AOR], 2.74; 95% CI, 1.94-3.88). The alert was associated with testing in the intervention group for reproductive health–related visits (AOR, 2.80; 95% CI, 1.70-4.62) and for other medical reasons (AOR, 2.94; 95% CI, 1.78-4.85). The alert did not affect screening in obstetrics-gynecology practices.

**Conclusions and Relevance:**

In this randomized clinical trial of chlamydia screening, an alert directed to medical assistants was associated with increased odds of a test ordered in primary care practices. This study’s results suggest that an alert could increase the proportion of women screened for chlamydia and reduce the reproductive sequelae of this common STD.

**Trial Registration:**

ClinicalTrials.gov Identifier: NCT03246815

## Key Points

**Question:**

Can an electronic medical record alert directed to medical assistants when preparing patients for their office visits increase screening for chlamydia among young women?

**Findings:**

In this randomized clinical trial of women aged 18 to 24 years with 7356 encounters in primary care and 10 672 in obstetrics-gynecology, an electronic medical record alert in primary care practices was associated with an increase in test orders compared with the control group. The alert did not affect testing in obstetrics-gynecology practices.

**Meaning:**

This study’s findings suggest that an automatic alert in the electronic medical record directed to medical assistants increases chlamydia screening among young women in primary care settings.

## Teaser

This randomized clinical trial investigates the effect of an electronic alert directed at medical assistants in a large health care system on chlamydia screening among women.

## Introduction

Four million chlamydia and 1.6 million gonorrhea infections are estimated to occur annually in the US.^1^ In women, these infections can cause pelvic inflammatory disease and infertility. Because chlamydia and gonorrhea infections are often asymptomatic, screening sexually active women 24 years and younger is recommended to prevent pelvic inflammatory disease and infertility.^2,3^ Unfortunately, the rates of screening for chlamydia in women in the US remain low.^4^

Access to care, practitioner awareness, and time constraints are among the many barriers to chlamydia screening.^5^ Preventive health is often overlooked during problem-focused visits. Innovative strategies are needed to facilitate screening young women. Computer-based alerts remind practitioners of recommended care that could be overlooked during an office visit, but too many alerts might lead to practitioners ignoring them, a phenomenon called alert fatigue.^6,7,8^ Interventions addressing care gaps are most successful when they are user-centered with limited impact on workflow. We investigated the effect of an electronic alert directed at medical assistants in a large health care system on chlamydia screening among women.

## Methods

### Study Design and Population

The STD Testing in Outpatient Practices (STOP STDs) Study was a pragmatic cluster randomized trial conducted in Western Pennsylvania from August 2017 to November 2019. We evaluated a medical assistant–facing electronic alert reminder aimed to increase chlamydia and gonorrhea screening among women receiving care at primary care and obstetrics-gynecology offices. The University of Pittsburgh institutional review board approved this study and granted a waiver of informed consent because otherwise the research would not have been able to be performed in this minimal risk study. The study follows the Consolidated Standards of Reporting Trials (CONSORT) reporting guideline. The trial protocol can be found in Supplement 1.

### Inclusion and Exclusion Criteria

**Figure 1.**
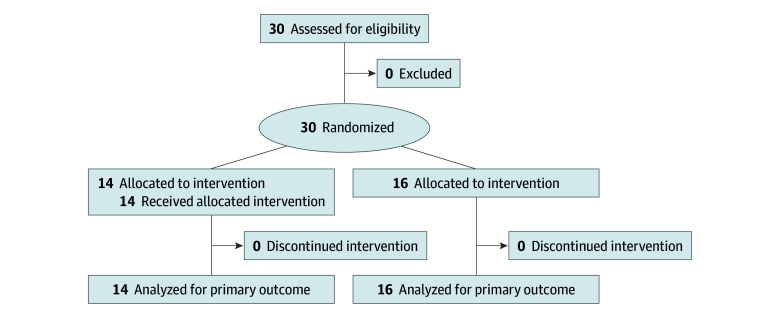
CONSORT Flow Diagram of the Progress Through the Randomized Clinical Trial of the 2 Study Groups

Offices from 3 large community-based, multisite group practices (2 primary care groups, consisting of internal medicine or family medicine specialties, and 1 obstetrics-gynecology group) owned by the University of Pittsburgh Medical Center (UPMC) were considered for participation. To ensure an adequate number of patients during site selection, primary care practices were eligible if they provided care to at least 200 females aged 16 to 24 years in the previous year. Practices were also characterized by practice location and racial distribution of patients as recorded in the electronic medical record (EMR), reflecting different chlamydia rates based on location and race. Race and ethnicity information was obtained from the EMR and is self-reported by patients. Race and ethnicity categories included Black, non-Hispanic; Hispanic; White, non-Hispanic; and other race, non-Hispanic (including non-Hispanic American Indian and Alaska Native, non-Hispanic Asian, non-Hispanic Native Hawaiian and Other Pacific Islander, and non-Hispanic other). Physician leaders at each practice were approached by one of the investigators (H.C.W.) to solicit participation. The study involved patients aged 18 to 24 who were female (as previously populated in the EMR) without a chlamydia test documented in the EMR in the prior 365 days. Practices not using UPMC’s EMR system were ineligible. Patients indicated as being pregnant in the EMR were excluded. Figure 1 shows the CONSORT flow diagram.

### Randomization

The primary care and obstetrics-gynecology practices were each randomized separately in a 1:1 ratio into intervention (alert) and control groups. Randomization was accomplished in the primary care practices by stratifying the practices according to urbanicity of the county where the practice was located (US Department of Agriculture Rural Urban Continuity Code [RUCC] = 1 vs RUCC ≠ 1) and by stratifying according to the percentage of the practice patient population of eligible women who were African American (above the median for African American women vs below the median for African American women). Practices in each of the resulting 4 stratification groups were randomized using a random-number generator to be intervention or control. In obstetrics-gynecology practices, randomization was performed by stratifying on the basis of the percentage of the practice patients who were African American only, due to all but 1 of the obstetrics-gynecology practices being in RUCC equal to 1.

### Intervention

The intervention was a real-time alert in the EMR that informed health care personnel whether their patient was eligible for chlamydia screening. Practices in the control group (usual care) did not receive the alert. The computer-generated alert was automatically created at the time of the visit for nonpregnant women aged 18 to 24 years who were eligible for chlamydia screening, which was defined as not having a chlamydia test order in the EMR within the past year. The alert was directed to medical assistants (unlicensed staff who perform tasks, such as taking vital signs) when preparing patients for their visit (known as rooming). The medical assistant could order a chlamydia and gonorrhea test through a link in the alert and obtain a urine sample or a self-collected vaginal swab. Orders were queued for clinician signature later in the visit. When tests were not ordered, the staff could indicate whether the patient declined, was not sexually active, or was screened elsewhere, and this response suppressed the alert in subsequent encounters for 30 days. If the medical assistant did not queue the orders, clinicians could still order tests outside the alert through their usual practice (eFigure in Supplement 2).

Before the study, one of the investigators (H.C.W.) met with staff at each practice randomized to the intervention, reviewing chlamydia and gonorrhea screening recommendations. The medical assistants were asked to inform patients that screening is routine and recommended by the practitioners, but they were not given a script or specific wording. Every 3 months, an investigator contacted staff at intervention practices to confirm that the alerts were received, but no other information was shared or offered.

### Outcomes

The primary outcome was the number of orders for chlamydia screening placed during eligible encounters. Test orders, rather than results, were chosen as the outcome because some laboratories do not interface with UPMC’s EMR system. Gonorrhea orders were not an outcome because they are typically performed alongside chlamydia testing. Test order rates were compared between the study groups separately for primary care and obstetrics-gynecology practices, accounting for repeated encounters within patients and clustering within practices. The secondary outcome was chlamydia orders placed during visits related to reproductive health or visits for other reasons. Two investigators (H.C.W. and G.S.F.) categorized each encounter based on *International Statistical Classification of Diseases and Related Health Problems, Tenth Revision (ICD-10) *diagnosis codes as reproductive or nonreproductive (eTable 1 in Supplement 2).

### Statistical Analysis

Deidentified reports from the EMR provided data for analysis. Race and ethnicity information was obtained from the EMR and is self-reported by patients. To address preintervention variation in chlamydia testing rates and estimate intervention effects as a differential pre-to-post change, we used constrained difference-in-differences (DiD) mixed-effects logistic regression with random intercepts for practice and patient to account for within-cluster correlation and repeated encounters within patients.^9,10,11^ Models included fixed effects for time and time × intervention interaction and were fit separately for primary care and obstetrics-gynecology practices. Because there were fewer than 50 clusters, 95% CIs and *P* values were based on a t-distribution with degrees of freedom = K − p, where K is the number of clusters and p is the number of cluster-level covariates.

Given 2 parallel cohort–specific analyses of the same end point, we also applied a Bonferroni correction to control the familywise error rate at 0.05 (α = .025 per comparison). The prestudy periods for primary care and obstetrics-gynecology practices were 619 and 690 days, respectively. The study was powered to detect a 10% change in screening rates between intervention and control groups with an α = .05. A 2-sided *P* < .05 was considered statistically significant. Data analysis was performed from August 2022 to July 2023. Analyses were performed using R software, version 4.4 (R Foundation for Statistical Computing).

## Results

The study was conducted in 16 primary care practices (8 in the intervention group and 8 in the control group) equally proportioned between metropolitan and nonmetropolitan locations. Six of 14 obstetrics-gynecology practices were randomized to the intervention group and 8 to the control group. Cluster-level intraclass correlation coefficients were 0.25 for primary care and 0.07 for obstetrics-gynecology practices.

### Primary Care Practices

Among primary care practices, 3770 eligible encounters occurred in the intervention practices and 3586 eligible encounters in the control practices. The median (IQR) age of patients was 20.1 (18.0-22.0) years in the intervention group and 20.3 (18.0-23.0) years in the control group (Table 1). In the intervention group, 261 (6.9%) were Black, non-Hispanic, 78 (2.1%) were Hispanic, 3206 (85.0%) were White, non-Hispanic, and 110 (2.9%) were other race, non-Hispanic. In the control group, 224 (6.2%) were Black, non-Hispanic, 42 (1.2%) were Hispanic, 3111 (86.8%) were White, non-Hispanic, and 94 (2.6%) were other race, non-Hispanic (Table 1). The intervention group had a higher proportion of patients who never smoked tobacco (standardized mean difference [SMD], 0.23) and who had commercial insurance (SMD, 0.21). All other patient characteristics were similar between groups (SMDs <0.10).

**Table 1.** Characteristics of Patients in Primary Care and Obstetrics-Gynecology Practices

Characteristic	No. (%) of patients^a^	
	Intervention (alert)	Control (usual care)
**Primary care practices**		
Total No.	3770	3586
Age, median (IQR), y	20 (18-22)	20 (18-23)
Insurance^b^		
Commercial or Medicare	2774 (73.6)	2295 (64.0)
Medicaid	874 (23.2)	1147 (32.0)
Other^c^	13 (0.3)	26 (0.7)
Marital status		
Single	3545 (94.0)	3345 (93.3)
Married or cohabited	135 (3.6)	182 (5.1)
Other^d^	89 (2.4)	54 (1.5)
Race and ethnicity		
Black, non-Hispanic	261 (6.9)	224 (6.2)
Hispanic	78 (2.1)	42 (1.2)
White, non-Hispanic	3206 (85.0)	3111 (86.8)
Other, non-Hispanic^e^	110 (2.9)	94 (2.6)
Tobacco use^b^		
Current smoker	271 (7.2)	339 (9.5)
Former smoker	153 (4.1)	208 (5.8)
Never smoker	2946 (78.1)	2444 (68.2)
**Obstetrics-gynecology practices**		
Total No.	5857	4815
Age, median (IQR), y	21 (18-23)	21 (18-23)
Insurance		
Commercial or Medicare	4356 (74.4)	3525 (73.2)
Medicaid	1408 (24.0)	1219 (25.3)
Other^c^	1 (0)	1 (0)
Marital status		
Single	5200 (88.8)	4392 (91.2)
Married or cohabited	472 (8.1)	301 (6.3)
Other^d^	179 (3.1)	122 (2.5)
Race and ethnicity		
Black, non-Hispanic	339 (5.8)	308 (6.4)
Hispanic	55 (0.9)	33 (0.7)
White, non-Hispanic	5121 (87.4)	4241 (88.1)
Other, non-Hispanic^e^	94 (1.6)	60 (1.2)
Tobacco use		
Current	568 (9.7)	484 (10.1)
Former	266 (4.5)	195 (4.0)
Never	4382 (74.8)	3744 (77.8)

^a^ Unless otherwise indicated. Totals might not add up due to missing data from the electronic medical record.

^b^ Standardized mean differences (SMDs) were calculated to assess baseline balance; in primary care practices, imbalance was greatest for tobacco use (never-smoker category; SMD = 0.23) and insurance type (maximum SMD = 0.21), whereas all characteristics had SMDs less than 0.10 in obstetrics-gynecology practices.

^c^ Other category in insurance included the auto category and additional unspecified categories.

^d^ Other category in marital status included the following subcategories: legally separated, divorced, or widowed and unknown or other categories.

^e^ Other, non-Hispanic category in race and ethnicity included the following subcategories: non-Hispanic American Indian and Alaska Native, non-Hispanic Asian, non-Hispanic Native Hawaiian and Other Pacific Islander, and non-Hispanic other.

**Figure 2.**
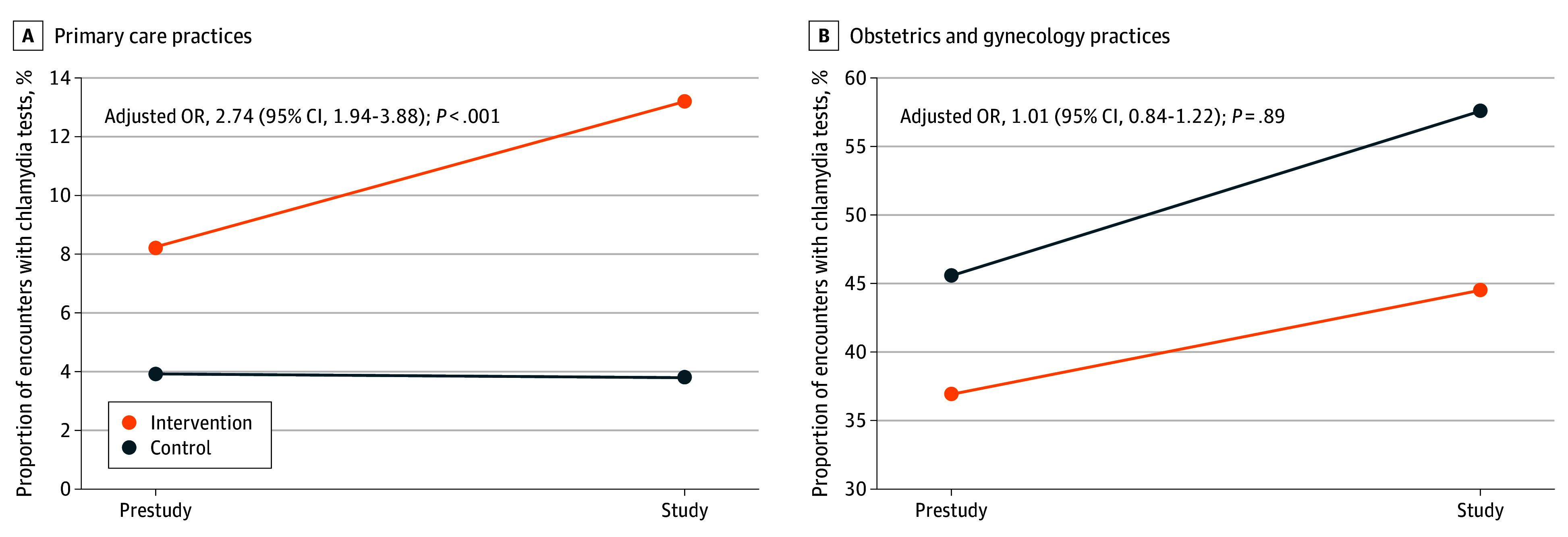
Line Graphs of Chlamydia Testing Among Primary Care and Obstetrics-Gynecology Practices A difference-in-differences analysis and mixed-effects logistic regression were used to account for preexisting differences in chlamydia testing rates and for the correlation of repeated encounters per patient among primary care (A) and obstetrics-gynecology practices (B).

During the study, chlamydia tests were ordered in 497 of 3770 encounters (13.2%) in the intervention group compared with 135 of 3586 encounters (3.8%) in the control group (Table 2). Assessing for the impact of baseline differences in testing, the number of encounters in the intervention group with a chlamydia test order significantly increased from 310 of 3762 (8.2%) before the study to 497 of 3770 (13.2%) during the study period (adjusted odds ratio [AOR], 2.72; 95% CI, 2.20-3.35; *P* < .001). In contrast, no significant change was observed in the control group; tests were ordered in 129 of 3291 encounters (3.9%) before the study and 135 of 3586 encounters (3.8%) during the study (AOR, 1.01; 95% CI, 0.73-1.39; *P* = .96). Using DiD analysis accounting for baseline differences in testing, the increase in the percentage of encounters with chlamydia test orders in the intervention group was statistically greater than that observed in the control group (AOR, 2.74; 95% CI, 1.94-3.88) (Figure 2).

**Table 2.** Chlamydia Test Orders Before and During the Study Period Among the Intervention and Control Groups

Characteristic	Test ordered, No. (%)		Total No. of visits	AOR (95% CI)^a^	*P* value^a^
	Yes	No			
**Primary care practices**					
Intervention group					
Prestudy period	310 (8.2)	3452 (91.8)	3762	1.0 [Reference]	NA
Study period	497 (13.2)	3273 (86.8)	3770	2.72 (2.20-3.35)	<.001
Control group					
Prestudy period	129 (3.9)	3162 (96.1)	3291	1.0 [Reference]	NA
Study period	135 (3.8)	3451 (96.2)	3586	1.01 (0.73-1.39)	.96
**Obstetrics-gynecology practices**					
Intervention group					
Prestudy period	919 (36.8)	1581 (63.2)	2500	1.0 [Reference]	NA
Study period	2599 (44.4)	3258 (55.6)	5857	1.99 (1.69-2.34)	<.001
Control group					
Prestudy period	1344 (45.6)	1605 (54.4)	2949	1.0 [Reference]	NA
Study period	2775 (57.6)	2040 (42.4)	4815	2.01 (1.71-2.36)	<.001

Abbreviations: AOR, adjusted odds ratio; NA, not applicable.

^a^ AORs and *P* values were estimated using mixed-effects logistic regression that accounted for the correlation of repeated encounters per patient.

We examined the electronic alert’s impact during visits for reproductive vs other health concerns (Table 3). In the intervention group, 646 of 3770 visits (17.1%) were for reproductive health compared with 535 of 3585 visits (14.9%) in the control group. Test orders increased from 192 of 783 (24.5%) to 216 of 646 (33.4%) in visits for reproductive health and from 118 of 2978 (4.0%) to 281 of 3124 (9.0%) in visits for other concerns. Chlamydia tests were more frequently ordered during reproductive health visits in both groups. Compared with the prestudy period, chlamydia test orders increased in the intervention group in both reproductive and nonreproductive health visits. Conversely, testing in the control group did not increase over time, regardless of visit reason. Compared with the control group, tests were more frequently ordered in the intervention group for visits related to reproductive health (AOR, 2.80; 95% CI, 1.70-4.62) and visits unrelated to reproductive health (AOR, 2.94; 95% CI, 1.78-4.85).

**Table 3.** Comparison of Chlamydia Test Orders According to Reason for Office Visit Among Primary Care Practices by Study Group

Characteristic	Test order in the prestudy, No. (%)		Total No. of visits	Test order in the study period, No. (%)		Total No. of visits	AOR (95% CI)^a^	*P* value^a^
	Yes	No		Yes	No			
**Reproductive reason**								
Intervention group	192 (24.5)	591 (75.5)	783	216 (33.4)	430 (66.6)	646	2.80 (1.70-4.62)	<.001
Control group	78 (15.8)	415 (84.2)	493	64 (12.0)	471 (88.0)	535	1.0 [Reference	NA
**Nonreproductive reason**								
Intervention group	118 (4.0)	2860 (96.0)	2978	281 (9.0)	2843 (91.0)	3124	2.94 (1.78-4.85)	<.001
Control group	51 (1.8)	2747 (98.2)	2798	71 (2.3)	2979 (97.7)	3050	1.0 [Reference]	NA

Abbreviations: AOR, adjusted odds ratio; NA, not applicable.

^a^ AORs and *P* values were estimated using mixed-effects logistic regression and difference-in-differences analyses that accounted for the correlation of repeated encounters per patient and preexisting differences in testing rates.

### Obstetrics-Gynecology Practices

During the study period, 5857 eligible encounters occurred in the intervention group and 4815 in the control group (Table 1). There were no differences in patient characteristics between the intervention and control groups (SMDs <0.10). During the study, chlamydia tests were ordered in 2599 of 5857 encounters (44.4%) in the intervention group compared with 2775 of 4815 encounters (57.6%) in the control group (AOR, 0.45; 95% CI, 0.39-0.53) (Table 2). Comparison of testing rates before and during the study explains this difference because before the study chlamydia tests were ordered less frequently in the intervention practices than in the controls (919 [36.8%] vs 1344 [45.6%]; AOR, 0.55; 95% CI, 0.46-0.64). During the study, testing increased in both the intervention and control groups: from 919 (36.8%) to 2599 (44.4%) in the intervention group (AOR, 1.99; 95% CI, 1.69-2.34) and from 1344 (45.6%) to 2775 (57.6%) in the control group (AOR, 2.01; 95% CI, 1.71-2.36). After adjusting for baseline differences, the electronic alert had no statistically significant effect on chlamydia testing among obstetrics-gynecology practices (AOR, 1.01; 95% CI, 0.84-1.22) (Figure 2).

### Use of the Electronic Alert

To understand the use and impact of the intervention, we examined the staff’s interaction with the alert (eTable 2 in Supplement 2). Among primary care practices, staff opened the order set linked to the alert in 426 of 3384 encounters (12.6%). Tests were more commonly ordered when the alert’s order screen was used than when it was not used (282 of 426 [66.2%] vs 181 of 2958 [6.1%]; AOR, 114.65; 95% CI, 27.10-484.05). Among 929 visits during which staff indicated the reason for not ordering the test, 677 patients (72.8%) declined, 175 (18.8%) denied sexual activity, and 77 (8.3%) reported they were tested elsewhere within the past year. Among 2029 encounters (60.0%) during which the alert’s order set was not used and staff did not indicate why tests were not queued, tests were ordered outside the alert in 144 visits (7.1%). Among obstetrics-gynecology offices, the alert was used for order entry in 1280 of 5720 visits (22.4%); tests were ordered in 1142 of 1280 encounters (89%) when the order set was opened compared with 1425 of 4404 encounters (32.1%) when it was not opened. Among the 3577 encounters (62.5%) for which staff did not indicate why the test was not ordered, a test was ultimately ordered in 1262 (35.3%).

### Cancellation of Chlamydia Test Orders

Because physicians or advanced practice clinicians might ultimately decide not to test, we assessed the number of canceled orders. In primary care practices, 57 of 489 tests (11.7%) ordered in the intervention group were subsequently canceled, similar to the number of those canceled before the study (25 of 304 [8.2%]; AOR, 2.50; 95% CI, 0.48-13.19; *P* = .24) and to the control group (12 of 132 [9.1%]; AOR, 1.05; 95% CI, 0.31-3.58; *P* = .93). Among obstetrics-gynecology practices, 589 of 2589 ordered tests (22.8%) were canceled by the end of the visit—a significantly higher amount than that observed before the study (51 of 906 [5.6%]; AOR, 7.06; 95% CI, 4.78-10.42; *P* < .001) and higher than the amount in the control group (202 of 2772 [7.3%]; AOR, 11.16; 95% CI, 7.28-17.10; *P* < .001).

### Detection of Sexually Transmitted Infections

Results were available for 455 of 552 chlamydia tests (82.4%) ordered in primary care practices. Nine infections (2.5%) were detected in 366 tests in the intervention group and 9 of 89 (10.1%) in the control group. One *Neisseria gonorrhoeae* infection was detected among 361 tests (0.3%) with available results in the intervention group, and none were detected among controls. In obstetrics-gynecology practices, 96 chlamydia infections were detected among 1904 tests (5.0%) in the intervention group and 104 of 2510 tests (4.1%) in the control group.

## Discussion

An EMR alert embedded into the visit workflow of medical assistants in primary care was associated with a statistically significant 2.74-fold increase in the odds that women were ordered a chlamydia test. As testing increased in visits for both reproductive and other health concerns, the alert can broaden opportunities for screening patients whenever they seek care. The alert had no significant impact in obstetrics-gynecology practices.

The US Preventive Services Task Force has recommended routine chlamydia and gonorrhea screening of sexually active females 24 years or younger for more than 2 decades; however, screening rates remain low.^2^ From 2011 to 2020, only half of sexually active females aged 16 to 24 years with commercial insurance were screened for chlamydia, with rates only slightly higher among women with Medicaid.^4^ Many barriers impact STD screening. Patients unaware of the importance of screening might not seek STD services.^12^ Stigma surrounding STDs is associated with lower screening rates.^13,14^ Clinicians with limited sexual health knowledge are less likely to recognize the need for screening.^15^ Limited time during visits, particularly when focusing on concerns unrelated to sexual health, hinders STD screening.^16^ Some practitioners might be unaware that urine or self-collected vaginal swabs enable testing without a pelvic examination.^17^

Practitioner-level interventions to increase chlamydia screening are only moderately effective.^18^ Many interventions are labor intensive, limiting widespread adoption. Routinizing screening as standard care and opt-out testing increase screening but require staff to remember to offer screening.^19,20^ Busy clinicians might benefit from real-time reminders; however, prompts in paper medical records have been unsuccessful.^21^ Clinical decision support systems, including clinician-facing electronic alerts generating patient-specific recommendations, are widely used to promote best practices.^22^ Unfortunately, a systematic review of point-of-care electronic alerts directed at physicians demonstrated only a modest effect, with a median care improvement rate of 4.2%.^23^ High numbers of alerts can desensitize physicians and cause alert fatigue, leading to ignoring or dismissing the alerts.^24^ Our intervention was directed at medical assistants, thereby avoiding burdening clinicians with additional tasks. The alert was designed for easy integration in practices. The EMR automatically identified eligible patients and was designed to be user-friendly for staff, containing brief messaging and a direct link for orders. We observed a 10-fold increase in orders when staff responded to the alert compared with when it was dismissed (66.2% vs 6.1%), demonstrating that the alert contributed to the increase in testing.

Although it was not surprising that testing in primary care practices was more frequent during reproductive health visits, the intervention was associated with increased orders regardless of the reason for the visit. Test orders increased from 24.5% to 33.4% in visits for reproductive health and from 4.0% to 9.0% in visits for other concerns. Baseline screening rates were low, which might reflect that screening was uncommon in primary care offices or that some patients did not require screening (eg, not sexually active) or were screened in non-UPMC facilities. Although the overall chlamydia screening rate of 13.2% seems suboptimal, the cumulative magnitude of the increase in screening could be substantial because there are an estimated 41 million visits annually by females aged 15 to 24 years to US primary care physicians.^25^ Clearly though, additional strategies are needed to achieve high screening rates.

The alert did not impact testing in obstetrics-gynecology practices. When the alert was not used, 32.1% of patients in obstetrics-gynecology practices were ordered tests compared with only 6.1% in primary care practices, likely representing obstetrician-gynecologists’ inherent focus on sexual care. The higher rate of cancelled tests by obstetrician-gynecologists might reflect familiarity with screening guidance. The alert’s impact might have been blunted in obstetrics-gynecology practices because many participating offices empower staff to preemptively place orders for preventive care (eg, breast and cervical cancer).

### Strengths and Limitations

Our study has several strengths. Randomization of practices reduced selection bias. By accounting for baseline differences in screening and DiD methods, biases were reduced in postintervention comparisons between the 2 study groups, and biases from comparisons over time that could reflect trends due to other causes linked to the decision to screen. Despite randomization, differences in baseline screening between intervention and control groups are likely due to the small number of practices in each group. The DiD method allowed for meaningful interpretation of the intervention, even with these baseline differences. The alert was designed for easy implementation, leveraging the EMR to automatically identify eligible patients. Unlike studies with constant investigator oversight, we provided staff with minimal instruction without scripting and did not monitor use of the alert or intervene during the study, mimicking real-life practice. Including metropolitan and nonmetropolitan practices enhanced the generalizability of our results.

The study also has some limitations. We could only capture test orders but not tests performed, and not all results were available because some laboratories are not linked to the EMRs. We assessed only chlamydia orders, assuming that screening typically includes gonorrhea, reflecting standard practice. We did not capture symptoms and could not discern differences in testing symptomatic patients from screening asymptomatic patients. Because most patients with chlamydia are asymptomatic, we inferred that most orders were placed for screening. We had incomplete information about why tests were not ordered because staff could dismiss the alert without explanation. The brief overview of STDs provided only to staff in the intervention group could have impacted testing; however, this is unlikely because tests were ordered more frequently when the alert was used. Because this study was conducted in western Pennsylvania, the findings might not be generalizable to other regions. We did not assess the alert’s acceptability among patients or staff. Although the study concluded 7 years ago, these results remain applicable today because chlamydia screening rates in the US have not appreciably increased.^4^

## Conclusions

In this randomized clinical trial, embedding an EMR alert into medical assistant workflows in primary care was associated with a 2.74-fold increase in the odds of chlamydia test ordering among women. The alert’s impact was greater than previously studied interventions aimed at improving chlamydia screening.^18^ Directing the alert to medical assistants enabled testing while freeing up clinicians to focus on other aspects of care. The alert was successful in primary care practices for visits related and unrelated to reproductive health, broadening the opportunity to screen, regardless of why patients seek care. This study’s results suggest than an electronic alert represents an important tool to improve chlamydia screening in young women.

## Notes

Supplement 1.
Trial Protocol

Supplement 2. **eFigure.** Flow Diagram of the Electronic Alert and Order Processes **eTable 1.** ICD-10 Codes for Encounters Related to Reproductive Care **eTable 2.** Use of the Electronic Alert and Chlamydia Test Orders Among Primary Care and Obstetrics/Gynecology Practice Staff

Supplement 3.
Data Sharing Statement
