# Supplementary material for: A Staff-Directed Electronic Medical Record Alert to Increase Chlamydia Screening: A Randomized Clinical Trial
Source: JAMA Netw Open. 2026 May 29;9(5):e2615360. doi: 10.1001/jamanetworkopen.2026.15360 (PMC13221685; doi:10.1001/jamanetworkopen.2026.15360)
Supplement: Supplement 1. — Trial Protocol [file jamanetwopen-e2615360-s001.pdf]

STD TESTING IN OUTPATIENT PRACTICES: The STOP STDs STUDY

Trial Registration: NCT03246815

Protocol Version: 2.0 August 2017

Funding Agency: The Centers for Disease Control and Prevention Foundation, Atlanta, GA

Principal Investigator: Harold C. Wiesenfeld, MD, CM
Professor
University of Pittsburgh School of Medicine
Department of Obstetrics, Gynecology and Reproductive Sciences
Magee-Womens Hospital of UPMC
300 Halket Street, Suite 2333
Pittsburgh, PA , 15213
wieshc@upmc.edu

The funding agency did not have a role in study design, data collection, data management, analysis, interpretation of data, or in manuscript writing or decision to submit the manuscript for publication

TABLE OF CONTENTS

1. Background and Rationale ……………………………………………………4
2. Study Objectives……………………………………………………………5
3. Trial Design…………………………………………………………………..5
4. Study Population……………………………………………………………5
5. Study Procedures…………………………………………………………..5
   5.1 Electronic Alert
   5.2 Study Arms
   5.3 Site Selection and Randomization
   5.4 Procedures in the Intervention Arm
   5.5 Procedures in the Control Arm
6. Microbiology Testing………………………………………………………..7
7. Data Collection and Management……………………………………...…8
8. Sample Size Calculation…………………………………………..……8
9. Data Analysis…………………………………………………………………8
10. Safety And Dissemination…………………………………………………..8
11. Costs And Payments…………………………………………………………9
12. Funding…………………………………………………………………………9
13. References……………………………………………………………………..9

PROTOCOL SUMMARY

Short Title: **STOP** (**S**TD **T**esting in **O**utpatient **P**ractices) **STDs STUDY**

Principal Investigator: Harold C. Wiesenfeld , M.C., C.M.

Study Population: Primary care practices and obstetrics and gynecology practices affiliated with the University of Pittsburgh Medical Center (UPMC) in Western Pennsylvania providing care to women 18-24 years old.

Study Design: A randomized pragmatic cluster trial of an alert in the electronic health record directed at medical assistants during the rooming in of patients to offer chlamydia and gonorrhea screening in primary care and obstetrics/gynecology (OBGYN) practices.

Primary care and OBGYN practices will separately be randomized to the intervention (electronic alert) and control groups (usual care). Medical assistants in practices randomized to the Intervention will receive a real-time alert reminder in the electronic medical record (EMR) to offer chlamydia and gonorrhea screening to patients likely eligible for screening during rooming in for office visits. In practices randomized to the control arm, no alert will be provided. The main outome of the study is chlamydia test orders, which will be compared between the intervention (alert) and control arms, separately for primary care practices and for obstetrics/gynecology practices.

1. Background and Rationale

Routine screening of sexually active young women for *Chlamydia trachomatis* and *Neisseria gonorrhoeae* is recommended in order to prevent the development of pelvic inflammatory disease and to reduce transmission of these sexually transmitted organisms.[1] Young women are disproportionately burdened by chlamydia and gonorrhea, and adolescent females have had the highest prevalence of infection. Based on estimates from national surveys conducted from 1999–2008, chlamydia prevalence among sexually active females aged 14–19 years was 6.8% overall and 16.2% among non-Hispanic black women.[2] Analysis of LabCorp chlamydia testing data from 2008-2010 found that chlamydia positivity ranged from 6.9%-10.7% among women aged 15-21 years with chlamydial symptoms, and 6.1%-9.6% among asymptomatic women.[3,4]

Sexually transmitted diseases are associated with adverse reproductive health outcomes and increased HIV transmission.[5] Chlamydial and gonococcal infections in women are often asymptomatic and when untreated can result in complications such as pelvic inflammatory disease (PID) with long term reproductive sequelae of infertility, ectopic pregnancy, and chronic pelvic pain.[6] As these infections are commonly asymptomatic in women, screening for these infections in at-risk individuals is important to enable earlier detection and treatment and the protection of reproductive health of women. Two randomized controlled studies found that chlamydia screening and treatment decreased the incidence of PID.[7,8]

As most women infected with chlamydia and gonorrhea are asymptomatic, screening programs have been key strategic components of prevention efforts. Despite national screening recommendations, screening rates among young women have been suboptimal.[4,9-13] Only 11% of 0.5 million sexually active women aged 15 years reported being tested for chlamydia in the prior year.[12] Among women aged 15-21 years, chlamydia testing occurred very infrequently at visits to pediatricians (< 1%), and in only 28% of visits to a physician where a Pap test was performed.[4] Several barriers to chlamydia screening have been identified. Taking a sexual history, particularly from younger patients is especially challenging. Providers may not recognize the need to screen at-risk patients. Many competing demands and priorities in an often brief clinical encounter can also be a barrier to STD screening, especially given the sensitive discussion about sexual behaviors that is necessary to identify those who should undergo screening. As such, novel strategies are urgently needed in order to overcome these barriers and increase screening rates for chlamydia and gonorrhea.

Interventions to increase chlamydia and gonorrhea screening directed to the providers have only been moderately effective. Clinical decision support systems, including clinician-facing electronic alerts that generate patient-specific recommendations, are widely used to promote best practices.[14] However, a systematic review of point-of-care electronic alerts directed at physicians yielded only a modest effect, with a median care improvement of only 4.2%.[15] The sheer number of alerts can desensitize a busy physician and cause alert fatigue, leading to ignoring or dismissing the alerts.[16] Medical assistants generally do not receive alerts about clinical care and therefore might be less likely to face alert fatigue. Medical assistants might be more likely to interact with alerts

than other clinicians as they work under the direction of medical leadership of their practices. Further, an intervention directed at medical assistants during rooming in of patients could avoid burdening clinicians with additional tasks.

2. Study Objectives

The hypothesis of this study is that proportion of women ordered chlamydia screening will be higher in practices randomized to an electronic alert directed at medical assistants during rooming in of patients for their office visit than in practices without the electronic alert. Our primary objective is to compare the test order rates for *C. trachomatis* between practices randomized to the electronic alert compared to practices operating under routine care, separately for primary care practices and OBGYN practices. The secondary outcome will be to determine the test order rate in visits for reproductive care compared to visits for other medical reasons.

3. **Trial Design:** Randomized pragmatic cluster trial

4. Study Population

Primary care practices will be recruited from two large primary care groups in the UPMC Health System. These groups contain family medicine and internal medicine practices. Obstetrics and gynecology practices affiliated with the Department of Obstetrics, Gynecology and Reproductive Sciences at UPMC will be recruited. Practices must be utilizing EPIC (Epic Systems Corporation, Verona, Wisconsin) as their EMR. Medical leaders at each site will be approached for their interest in participating in this randomized trial. Practices whose leaders agree to participate will be potentially eligible for participation.

5. Study Procedures

5.1 Electronic Alert

An automated alert will be developed with the assistance of the UPMC informatics group. The electronic alert will be generated at the time of the office visit for practices in the intervention arm if the following criteria are met (all must be present):

1. Female patients (defined as the designated legal sex previously populated in the demographic section of the electronic health record)
2. Age 18-24
3. Non pregnant (defined as the absence of an active Pregnancy Episode in the EMR)
4. No order for a chlamydia test in the EMR in the preceding 365 days

The alert contains brief information about the importance of chlamydia and gonorrhea screening to women's health. The alert will be directed at the medical assistant during the beginning of the patient's visit, typically during the rooming process as patients are being prepared for the visit with the provider. The alert will inform the medical staff that chlamydia and gonorrhea screening is recommended for sexually active women age 24 and younger and will provide an option to place an order. A hyperlink button linked to the order entry will be in the alert, with chlamydia and gonorrhea orders on display for ordering by the MA for later signature by the clinicians.

Wording of the Alert: The electronic alert will contain the following wording:

> "Routine annual chlamydia and gonorrhea screening is recommended for all sexually active women age 24 and younger. Please use SmartSet to order screening by urine sample or self-collected vaginal swab if appropriate, or indicate the reason for not placing orders today"
>
> TABS: OPEN SMARTSET DO NOT OPEN SMARTSET
>
> Acknowledge Reason (tabs):
>
> NOT SEXUALL ACTIVE PATIENT DECLINED SCREENED IN LAST YEAR

5.2 Study Arms

Intervention Arm (Electronic Alert) of Office Practices: Sexually active women 18-24 years old seeking care at practices randomized to the intervention group will be offered screening by the office staff (medical assistant) at the time of the visit. An automated alert generated in the EMR will direct the staff member to offer chlamydia and gonorrhea screening to eligible women.

Control Arm of Office Practices: Screening for chlamydia and gonorrhea will be at the discretion of the physicians or advanced-care providers per their usual care. The electronic alert will not be active in these practices.

5.3 Site Selection and Randomization

Practices will be categorized by type of practice/specialty [primary care and OBGYN], location (metropolitan /urban vs. rural), and proportion of patients categorized in the EMR as African American. Primary care practices must have provided care to at least

200 females aged 16-24 in the previous year to be eligible for participation. Practices will then be matched and randomized to the intervention or control groups. Details on randomization are included in the Statistical Analysis Plan.

5.4 Procedures in the Intervention Arm

Staff at the office practices randomized to the intervention group will undergo a brief 30 minute educational session by the principal investigator that will include a review of the screening guidelines and the importance of chlamydia and gonorrhea screening for women's reproductive health. During that session, the staff at each practice, typically medical assistants will be advised to offer screening without coercion. Staff will have the option of ordering a test by urine sample or self-collected vaginal swabs according to practice and patient preference. Staff will be informed that screening is typically performed at no cost to the patient if the patient has medical insurance. The EMR alert will include a link that will direct the staff to the order entry section of the record, with options for chlamydia and gonorrhea tests embedded into the order screen. The order will be placed and held (also known as "pended") for later signature by the physician or advanced practice provider.

Staff leaders (e.g. office managers) at the Intervention sites will be contacted by the research assistant by telephone at three month intervals. The sole purpose of these calls is to verify that the staff are receiving the alerts, thereby confirming that the EMR alerts are functioning properly. Office practices will have the opportunity to raise any safety concerns at that time. No additional educational training or information will be shared by the research staff member during these contacts.

5.5 Procedures in the Control Arm

Office practices randomized to the control arm (usual care) will not have the alert activated in their EMR. The staff will not receive an educational session, nor will they be contacted during the study period.

6. Microbiologic Testing

Urine or self-collected vaginal swab samples (at the discretion of the office practice/staff and the patient) will be obtained for *C. trachomatis* and *N. gonorrhoeae* testing. Samples will be sent per office routine to their preferred microbiology laboratories, often those affiliated with UPMC (UPMC labs and Quest Laboratories). Testing will typically be performed using the APTIMA platforms per the routine of the labs in Western Pennsylvania. Costs of these tests would be charged to the patient's insurance provider, and insured patients will not have any cost for the test as chlamydia and gonorrhea screening are recommended preventive care and therefore are covered services. The results of screening will be reported back into the EMR for review by the clinical team per usual routine if the laboratory interfaces electronically with UPMC's EMR. Infections will be treated per office/provider routine.

7. Data Collection and Management

The research informatics team at UPMC (CARE, see below) will capture pertinent clinical data in EMR for women attending in both the intervention and control practices. They will provide demographic and clinical information for data analyses that will include age, race, reason for visit, clinical services performed, chlamydia and gonorrhea test orders, and test results (if test results are available). All data will be de-identified prior to transfer to the investigators and to the CDC for data analyses.

The Center for Assistance in Research using eRecord (CARE) provides facilitated managed, compliant and secure access to the UPMC Electronic Health Record for researchers. CARe utilizes the EMRs and databases in use by UPMC. EMRs are currently deployed in both inpatient and outpatient clinics throughout the health system and provide a wealth of information for investigators. EpicCare is the EMR product in use in ambulatory clinics and outpatient offices affiliated with UPMC. The data contained in the EMRs are maintained behind a secure firewall on servers within the UPMC data center.

8. Sample Size Calculation

Assuming that the screening rate in the usual care group is 30% and in the opt-out group it will be 35%, a study recruiting 1882 patients win the usual care group and 1882 patients in the opt-out group will result in 90% power to detect a significant difference in the proportion of women screened for chlamydia at a two sided .05 significance level. As such we propose to recruit at least 2000 patients in the opt-out group and 2000 patients in the usual care group.

If the baseline screening is closer to the 2011-2012 average commercial (non-Medicaid) screening rate of sexually active women (40% in 16-20 year olds and 47% in 21-24 year olds) we would need to enroll 898 women ages 16-20 and 228 women ages 21-24 in order to demonstrate a 20% increase in screening at 90% power. The proposed study would provide enough power to examine both of these age groups.

9. **Data Analysis** – See Statistical Analysis Plan

10. Safety And Dissemination

Existing patients of UPMC will be part of this study. The study outcome (chlamydia and gonorrhea screening) is standard of care in the United States. Therefore there are no additional safety concerns to patients regarding the offer of a recognized preventive health screening using urine or swab collection for chlamydia and gonorrhea. Any concerns raised by the clinical staff at the practices during quarterly calls that are related to the conduct of this study will be reviewed by the Principal Investigator during monthly study meetings or at an earlier time if appropriate. Privacy of all patients will be

ensured by the use of an honest broker system that will protect the identity of each patient from the UPMC and the CDC investigators. Data transmitted to the CDC for analysis will be verified as de-identified before transmittal. Names and personal identifiers of physicians will not be collected, ensuring the privacy of the medical staff at each site. As the intervention is considered routine clinical practice (STD screening) we do not anticipate major safety issues during this study.

Findings of the project will be shared with the leadership at each office practice that participated in the study. Results will also be prepared for presentation at scientific meetings and for publication in peer-reviewed scientific journals.

11. Costs And Payments

There are no costs or payments to patients or office practices as the study examines screening as part of standard clinical care.

12. Funding

Funding for this investigator-initiated study is provided by the Centers for Disease Control and Prevention Foundation.

13. References

1. U. S. Preventive Services Task Force. Screening for chlamydia and gonorrhea: US Preventive Services Task Force Recommendation Statement. Jama 2021;326(10):949–956. DOI: 10.1001/jama.2021.14081.
2. Centers for Disease C, Prevention. CDC Grand Rounds: Chlamydia prevention: challenges and strategies for reducing disease burden and sequelae. MMWR Morb Mortal Wkly Rep 2011;60(12):370–3. (https://www.ncbi.nlm.nih.gov/pubmed/21451447).
3. Hoover KW, Tao G, Nye MB, Body BA. Suboptimal adherence to repeat testing recommendations for men and women with positive Chlamydia tests in the United States, 2008-2010. Clin Infect Dis 2013;56(1):51–7. DOI: 10.1093/cid/cis771.
4. Hoover KW, Leichliter JS, Torrone EA, et al. Chlamydia screening among females aged 15-21 years--multiple data sources, United States, 1999-2010. MMWR Suppl 2014;63(2):80–8. (https://www.ncbi.nlm.nih.gov/pubmed/25208262).
5. Fleming DT, Wasserheit JN. From epidemiological synergy to public health policy and practice: the contribution of other sexually transmitted diseases to sexual transmission of HIV infection. Sex Transm Infect 1999;75(1):3–17.

6. Westrom L, Joesoef R, Reynolds G, Hagdu A, Thompson SE. Pelvic inflammatory disease and fertility. A cohort study of 1,844 women with laparoscopically verified disease and 657 control women with normal laparoscopic results. Sex Transm Dis 1992;19(4):185–92.
7. Scholes D, Stergachis A, Heidrich FE, Andrilla H, Holmes KK, Stamm WE. Prevention of pelvic inflammatory disease by screening for cervical chlamydial infection. N Engl J Med 1996;334(21):1362–1366.
8. Oakeshott P, Kerry S, Aghaizu A, et al. Randomised controlled trial of screening for Chlamydia trachomatis to prevent pelvic inflammatory disease: the POPI (prevention of pelvic infection) trial. BMJ 2010;340:c1642 doi:10.1136/bmj.c1642.
9. Eugene JM, Hoover KW, Tao G, Kent CK. Higher yet suboptimal chlamydia testing rates at community health centers and outpatient clinics compared with physician offices. Am J Public Health 2012;102(8):e26–9. DOI: 10.2105/AJPH.2012.300744.
10. Hoover K, Tao G. Missed opportunities for chlamydia screening of young women in the United States. Obstet Gynecol 2008;111(5):1097–102. DOI: 10.1097/AOG.0b013e31816bbe9b.
11. Hoover K, Tao G, Kent C. Low rates of both asymptomatic chlamydia screening and diagnostic testing of women in US outpatient clinics. Obstet Gynecol 2008;112(4):891–8. DOI: 10.1097/AOG.0b013e318185a057.
12. Tao G, Hoover KW, Leichliter JS, Peterman TA, Kent CK. Self-reported Chlamydia testing rates of sexually active women aged 15-25 years in the United States, 2006-2008. Sex Transm Dis 2012;39(8):605–7. DOI: 10.1097/OLQ.0b013e318254c837.
13. Tao G, Hoover KW, Kent CK. Chlamydia testing patterns for commercially insured women, 2008. American journal of preventive medicine 2012;42(4):337–41. DOI: 10.1016/j.amepre.2011.11.013.
14. Bright TJ, Wong A, Dhurjati R, et al. Effect of clinical decision-support systems: a systematic review. Ann Intern Med 2012;157(1):29–43. DOI: 10.7326/0003-4819-157-1-201207030-00450.
15. Shojania KG, Jennings A, Mayhew A, Ramsay C, Eccles M, Grimshaw J. Effect of point-of-care computer reminders on physician behaviour: a systematic review. CMAJ 2010;182(5):E216–25. DOI: 10.1503/cmaj.090578.
16. US Department of Health and Human Services. Alert Fatigue. PSNet [internet]. Rockville (MD): Agency for Healthcare Research and Quality, US Department of Health and Human Services. 2019.

STOP STDs Statistical Analysis Plan (SAP)

Randomization

The primary care and obstetrics/gynecology practices were each randomized separately in a 1:1 ratio into intervention (alert) or control groups. Randomization was accomplished in the primary care practices by stratifying the practices according to urbanicity of the county where the practice was located (US Department of Agriculture Rural Urban Continuity Code (RUCC) = 1 versus RUCC ≠ 1) and by stratifying according to the percentage of the practice patient population of eligible women who were African-American (above the median for African-American women versus below the median for African-American women). Practices in each of the resulting four stratification groups were randomized using a random number generator to be intervention or control. In obstetrics/gynecology practices, randomization was done by stratifying on the basis of the percentage of the practice patient who were African-American only, due to all but 1 of the obstetrics/gynecology practices being in RUCC = 1.

Statistical Methods

In this study, we will employ the following statistical methods to analyze the impact of a real-time alert system on chlamydia screening orders:

- **Baseline Characteristics:** Baseline characteristics will be summarized by study arm within each cohort (primary care and obstetrics/gynecology). Between-arm differences may be evaluated using 2-sided hypothesis tests ($\alpha = 0.05$); however, P values will not be presented in Table 1. As a supplementary, sample size–independent measure,

standardized mean differences (SMDs) may also be calculated to aid interpretation of the magnitude of baseline differences and, if used, will be summarized briefly in the text.

- **Constrained Difference-in-Differences Mixed Effects Logistic Regression (Primary Approach)**: We will estimate the intervention effect using a constrained difference-in-differences mixed-effects logistic regression model with a logit link. The model will include fixed effects for time period and the time-by-intervention interaction to compare pre-to-post changes between intervention and control practices. Random intercepts for practice (cluster) and patient will be included to account for within-cluster correlation and repeated encounters within patients. Models will be fit separately for primary care and obstetrics/gynecology practices. We will also report the intraclass correlation coefficient (ICC) to quantify the within-cluster correlation in the outcome, which is relevant for interpreting clustering and the precision of cluster-randomized estimates.
- **Small-Sample Inference for Cluster Randomized Designs**: Because the number of randomized clusters is fewer than 50, 95% confidence intervals and P values will be constructed using a t distribution with degrees of freedom (df) equal to $K - p$, where K is the number of clusters, and p is the number of cluster-level covariates included in the model.

Data Handling

Each study population comprises multiple datasets that will undergo a systematic cleaning process based on pre-defined eligibility criteria, provider type, encounter type, department id, and date.

Datasets will be merged using Encounter ID, Patient ID, and a unique identifier generated by combining Patient ID and Date. This approach is necessary due to the varying ID structures across datasets; for instance, the demographic dataset includes only Patient ID, while other datasets contain both Patient ID and Encounter ID. In cases where Encounter ID is missing, the combination of Patient ID and Date will be utilized to maximize the use of available data.

To ensure data integrity, a manual examination will be conducted to address other data-related issues, including duplication and inconsistencies. Intermediate checks will involve assessing missing values and verifying data consistency, while final checks will evaluate the completeness and accuracy of the datasets. We will also document all data handling procedures to ensure transparency and reproducibility.

Primary and Secondary Analyses

The intervention consists of a real-time alert in the electronic medical record that will notify healthcare personnel when a non-pregnant woman aged 18-24 is eligible for chlamydia screening, defined as having no test order in the past year.

- **Primary Analysis**: The primary outcome will be the number of chlamydia screening orders placed during encounters. We will compare test order rates between intervention and control groups in primary care and obstetrics/gynecology practices.
- **Secondary Analysis**: The secondary analysis will evaluate the impact of the electronic alert on screening rates during reproductive health visits versus other visit types.

For each primary and secondary outcome, we will report the intervention effect as an odds ratio with a 95% confidence interval and P value (see Statistical Methods).

Statistical Software

All analyses will be conducted using R (version 4.4; R Foundation for Statistical Computing, Vienna, Austria), with appropriate packages as needed.

Significance Levels

The familywise significance level will be set at $\alpha = 0.05$ (2-sided). Because the primary endpoint will be evaluated in 2 parallel cohorts (primary care and obstetrics/gynecology practices), we will apply a Bonferroni correction, using $\alpha = 0.025$ per cohort-specific primary comparison. For secondary outcomes, hypothesis tests will be interpreted using a 2-sided significance threshold of $\alpha = 0.05$ unless otherwise specified.
