# Supplementary material for: A Staff-Directed Electronic Medical Record Alert to Increase Chlamydia Screening: A Randomized Clinical Trial
Source: JAMA Netw Open. 2026 May 29;9(5):e2615360. doi: 10.1001/jamanetworkopen.2026.15360 (PMC13221685; doi:10.1001/jamanetworkopen.2026.15360)
Supplement: Supplement 2. — eFigure. Flow Diagram of the Electronic Alert and Order Processes eTable 1. ICD-10 Codes for Encounters Related to Reproductive Care eTable 2. Use of the Electronic Alert and Chlamydia Test Orders Among Primary Care and Obstetrics/Gynecology Practice Staff [file jamanetwopen-e2615360-s002.pdf]

Supplemental Online Content

Wiesenfeld HC, Hong J, Xu T, et al. A staff-directed electronic medical record alert to increase chlamydia screening: a randomized clinical trial. *JAMA Netw Open*. 2026;9(5):e2615360. doi:10.1001/jamanetworkopen.2026.15360

eFigure. Flow Diagram of the Electronic Alert and Order Processes

eTable 1. ICD-10 Codes for Encounters Related to Reproductive Care

eTable 2. Use of the Electronic Alert and Chlamydia Test Orders Among Primary Care and Obstetrics/Gynecology Practice Staff

This supplemental material has been provided by the authors to give readers additional information about their work.

© 2026 Wiesenfeld HC et al. *JAMA Network Open*.

eFigure. Flow Diagram of the Electronic Alert and Order Processes

MEDICAL ASSISTANT ORDER PROCESS

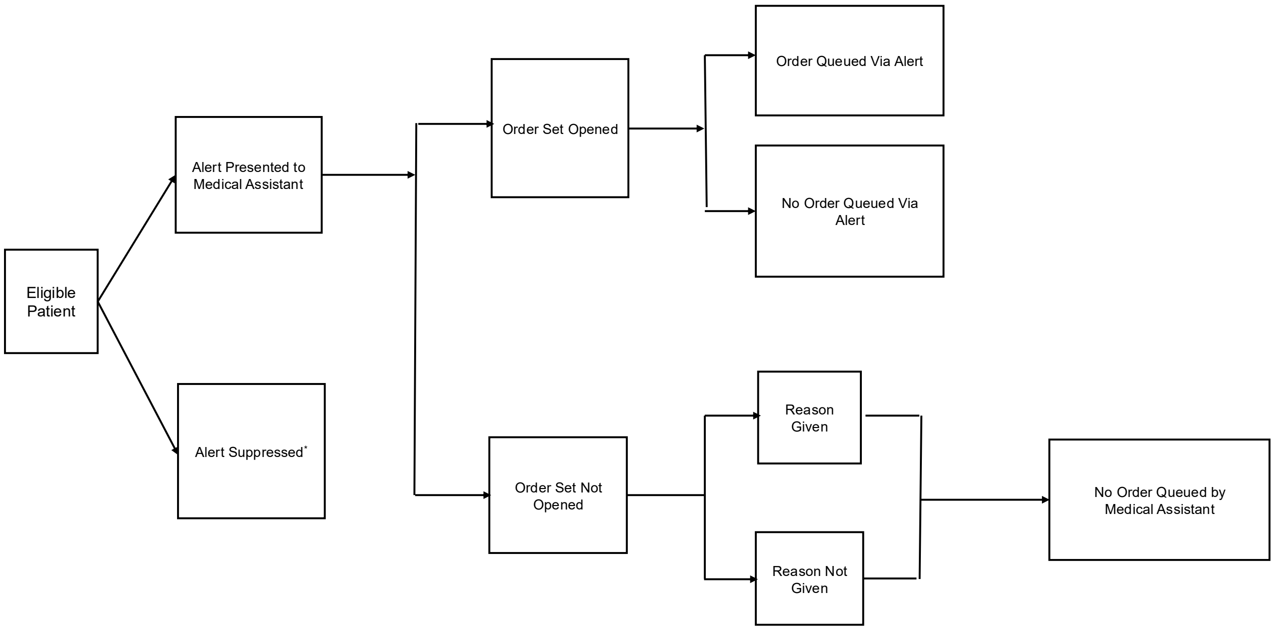

* Alert is suppressed if screening declined through an alert issued in an encounter in the prior 30 days

PROVIDER ORDER PROCESS

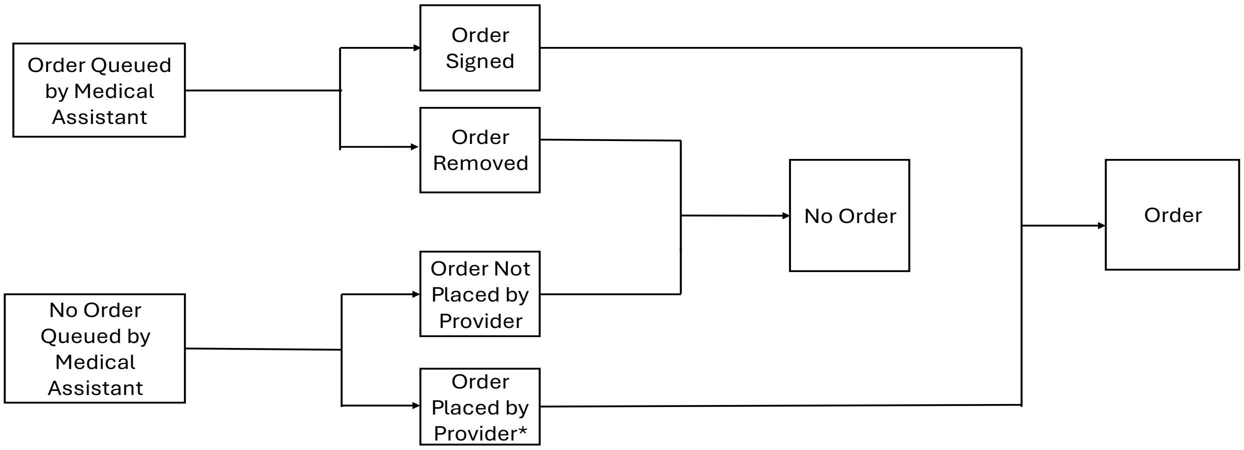

* Provider placed order per their routine practice (not via the orderset linked to the alert)

© 2026 Wiesenfeld HC et al. *JAMA Network Open*.

eTable 1. ICD-10 Codes for Encounters Related to Reproductive Care

ICD-10	NAME
A54.00	Gonococcal infection (acute) of lower genitourinary tract
A56	Chlamydia vaginitis/cervicitis
A59.03	Trichomonal cystitis
A59.9	Trichomonas vaginalis infection
A60.00	Genital herpes simplex, unspecified site
A60.04	Herpes simplex vulvovaginitis
A60.9	HSV (herpes simplex virus) anogenital infection
A64	STD (female)
A74.9	Chlamydia infection
B00.1	Herpes simplex labialis
B00.2	Primary HSV infection with gingivostomatitis
B00.89	Recurrent gingivostomatitis due to herpes simplex
B00.9	HSV (herpes simplex virus) infection
B27.00	Gammaherpesviral mononucleosis without complication
B97.7	HPV (human papilloma virus) infection
F52.9	Sexual dysfunction in females
F64.0	Transsexualism
IMO0002	Sexual assault by bodily force by person unknown to victim
R87.610	ASCUS of cervix with negative high risk HPV
R87.610, R87.810	ASCUS with positive high risk HPV cervical
T74.21XA	Sexual assault of adult, initial encounter
T74.21XD	Sexual assault of adult, subsequent encounter
T74.21XS	Sexual assault of adult, sequela
T76.22XS	Suspected victim of sexual abuse in childhood, sequela
Z11.51	Screening for HPV (human papillomavirus)
Z20.2	Exposure to sexually transmitted disease (STD)
Z23	Need for HPV vaccination
Z62.810	History of sexual abuse in childhood
Z62.810	Personal history of sexual abuse in childhood
Z71.1	Concern about STD in female without diagnosis
Z71.89	Counseling for HPV (human papillomavirus) vaccination
Z72.51	High risk sexual behavior
Z86.19	History of other infectious diseases
Z87.898	History of sexual violence
Z91.89	At risk for sexually transmitted disease due to unprotected sex
A60.04	Ulceration, vulva, herpetic
A63.0	Condyloma acuminata
N72	Cervicitis

© 2026 Wiesenfeld HC et al. *JAMA Network Open*.

N73.0	PID (acute pelvic inflammatory disease)
N75.1	Bartholin's gland abscess
N76.0	Vaginitis
N76.1	Subacute and chronic vaginitis
N76.2	Acute vulvitis
N76.4	Abscess, vulva
N76.5	Vaginal ulcer
N76.6	Genital ulcer, female
N80.9	Endometriosis
N83.201	Ovarian cyst (s)
N87.0	Dysplasia of cervix, low grade (CIN 1)
N87.9	Cervical dysplasia
N88.9	Abnormal cervix finding
N89.8	Vaginal discharge or itch or irritation
N90.7	Labial cyst
N90.89	Labial Lesion
N91.0	Delayed menses
N91.1	Amenorrhea, secondary
N91.2	Amenorrhea
N91.3	Primary oligomenorrhea
N91.4	Secondary oligomenorrhea
N91.5	Oligomenorrhea, unspecified type
N92.0	Excessive or frequent menstruation
N92.1	Metrorrhagia
N92.2	Excessive menstruation at puberty
N92.3	Intermenstrual bleeding
N92.4	Excessive bleeding in premenopausal period
N92.6	Irregular menses
N93.0	Bleeding after intercourse
N93.8	Dysfunctional uterine bleeding
N93.9	Abnormal uterine bleeding (AUB)
N94.0	Mittelschmerz
N94.10	Dyspareunia, female
N94.2	Vaginismus
N94.3	Premenstrual syndrome
N94.4	Primary dysmenorrhea
N94.6	Dysmenorrhea
N94.810	Vulvar vestibulitis
N94.819	Vulvodynia, unspecified
N94.89	Pelvic congestive syndrome
N94.9	Cervical motion tenderness
N97.0	Anovulation
N97.9	Infertility, female

© 2026 Wiesenfeld HC et al. *JAMA Network Open*.

O02.0	Complete molar pregnancy
O03.9	Miscarriage
O09.899	H/O preterm delivery, currently pregnant
R87.610	Atypical squamous cells of undetermined significance (ASCUS) on Papanicolaou smear of cervix
R87.612	Low grade squamous intraepithelial lesion on cytologic smear of cervix (LGSIL)
R87.613	HSIL (high grade squamous intraepithelial lesion) on Pap smear of cervix
R87.619	Abnormal cervical Papanicolaou smear, unspecified abnormal pap finding
R87.620	Atypical squamous cell changes of undetermined significance (ASCUS) on vaginal cytology
Z01.411	Encounter for gynecological examination with abnormal finding
Z01.419	Encounter for gynecological examination without abnormal finding
Z30.011	Encounter for initial prescription of contraceptive pills
Z30.012	Family planning, emergency contraceptive counseling and prescription
Z30.013	Encounter for initial prescription of injectable contraceptive
Z30.014	Encounter for initial prescription of intrauterine contraceptive device (IUD)
Z30.015	Encounter for initial prescription of vaginal ring hormonal contraceptive
Z30.016	Encounter for initial prescription of transdermal patch hormonal contraceptive device
Z30.016	Encounter for prescription for transdermal contraceptive
Z30.017	Insertion of implantable subdermal contraceptive
Z30.018	Encounter for initial prescription of other contraceptives
Z30.019	Encounter for female birth control
Z30.02	Encounter for counseling and instruction in natural family planning to avoid pregnancy
Z30.09	Encounter for counseling regarding contraception
Z30.40	Encounter for surveillance of contraceptives
Z30.41	Encounter for surveillance of contraceptive pills
Z30.42	Encounter for surveillance of injectable contraceptive
Z30.430	Encounter for insertion of intrauterine contraceptive device
Z30.431	Encounter for routine checking of intrauterine contraceptive device (IUD)
Z30.432	Encounter for IUD removal
Z30.433	Encounter for IUD removal and reinsertion
Z30.44	Encounter for surveillance of vaginal ring hormonal contraceptive device
Z30.45	Encounter for surveillance of transdermal patch hormonal contraceptive device
Z30.46	Encounter for surveillance of implantable subdermal contraceptive
Z30.49	Encounter for surveillance of other contraceptive
Z30.8	Encounter for other contraceptive management
Z30.9	Encounter for contraceptive management, unspecified type
Z31.41	Encounter for fertility testing
Z31.69	Encounter for preconception consultation
Z31.69	Infertility counseling
Z31.9	Infertility management
Z85.41	History of cervical cancer
Z87.42	History of other diseases of the female genital tract
Z97.5	IUD (intrauterine device) in place

© 2026 Wiesenfeld HC et al. *JAMA Network Open*.

eTable 2. Use of the Electronic Alert and Chlamydia Test Orders Among Primary Care and Obstetrics/Gynecology Practice Staff

Characteristic	No. (%) Test ordered Yes	No	Total	Adjusted odds ratio[a] (95 CI)	*P* value[a]
**Primary care practices**					
Order set opened	282 (66.2)	144 (33.8)	426	114.65 (27.10-485.05)	< .0001
Order set not opened	181 (6.1)	2777 (93.9)	2958	Ref	
Reason provided	37 (4.0)	892 (96.0)	929	-	
Reason not provided	144 (7.1)	1885 (92.9)	2029	-	
**Obstetrics/gynecology practices**					
Order set opened	1142 (89.2)	138 (10.8)	1280	42.74 (27.71-65.92)	< .0001
Order set not opened	1425 (32.1)	3015 (67.9)	4440	Ref	
Reason provided	163 (18.9)	700 (81.1)	863	-	
Reason not provided	1262 (35.3)	2315 (64.7)	3577	-	

[a] Order set opened versus order set not opened. Adjusted odds ratios and *P* values were estimated using mixed effects logistic regression that accounted for the correlation of repeated encounters per patient.
