# Supplementary material for: A Staff-Directed Electronic Medical Record Alert to Increase Chlamydia Screening: A Randomized Clinical Trial
Source: JAMA Netw Open. 2026 May 29;9(5):e2615360. doi: 10.1001/jamanetworkopen.2026.15360 (PMC13221685; doi:10.1001/jamanetworkopen.2026.15360)
Supplement: Supplement 3. — Data Sharing Statement [file jamanetwopen-e2615360-s003.pdf]

Data Sharing Statement

Wiesenfeld. A Staff-Directed Electronic Medical Record Alert to Increase Chlamydia Screening. *JAMA Netw Open*. Published May 29, 2026. doi:10.1001/jamanetworkopen.2026.15360

Data
Additional Information: ClinicalTrials.gov ID NCT03246815
https://clinicaltrials.gov/study/NCT03246815?term=NCT03246815&rank=1
Data available: Yes
Data types: Deidentified participant data
How to access data: Via request to corresponding author (Wiesenfeld): wieshc@upmc.edu
When available: With publication

Supporting Documents
Document types: None

Additional Information
Who can access the data: To researchers whose proposed use of the data has been approved by UPMC
Types of analyses: For additional analyses including metaanalysis
Mechanisms of data availability: Data will be mae available without investigator support, after approval of the request (by UPMC) and with a signed data access agreement
